# Sense of coherence predicts post-myocardial infarction trajectory of leisure time physical activity: a prospective cohort study

**DOI:** 10.1186/1471-2458-11-708

**Published:** 2011-09-19

**Authors:** Vicki Myers, Yaacov Drory, Yariv Gerber

**Affiliations:** 1 Department of Epidemiology and Preventive Medicine, School of Public Health, Sackler Medical School, Tel Aviv University, Israel; 2 Department of Rehabilitation, Sackler Medical School, Tel Aviv University, Israel

**Keywords:** myocardial infarction, physical activity, secondary prevention, sense of coherence, psychosocial factors, cohort study

## Abstract

**Background:**

Physical activity confers a survival advantage after myocardial infarction (MI), yet the majority of post-MI patients are not regularly active. Since sense of coherence (SOC) has been associated with health outcomes and some health behaviours, we investigated whether it plays a role in post-MI physical activity.

We examined the predictive role of SOC in the long-term trajectory of leisure time physical activity (LTPA) after MI using a prospective cohort design.

**Methods:**

A cohort of 643 patients aged ≤ 65 years admitted to hospital in central Israel with incident MI between February 1992 and February 1993 were followed up for 13 years. Socioeconomic, clinical and psychological factors, including SOC, were assessed at baseline, and LTPA was self-reported on 5 separate occasions during follow-up. The predictive role of SOC in long-term trajectory of LTPA was assessed using generalized estimating equations.

**Results:**

SOC was consistently associated with engagement in LTPA throughout follow-up. Patients in the lowest SOC tertile had almost twice the odds (odds ratio,1.99; 95% confidence interval,1.52-2.60) of decreasing their engagement in LTPA as those in the highest tertile. A strong association remained after controlling for disease severity, depression, sociodemographic and clinical factors.

**Conclusion:**

Our evidence suggests that SOC predicts LTPA trajectory post-MI. Assessment of SOC can help identify high-risk MI survivors, who may require additional help in following secondary prevention recommendations which can dramatically improve prognosis.

## Background

Physical activity confers a survival advantage on post-myocardial infarction (MI) patients [1-4]. Regularly active MI survivors were shown to have approximately half the risk of dying compared with inactive patients, irrespective of pre-MI habits [5]. Despite this clear advantage, a minority of post-MI patients are regularly active [5,6]. It remains unclear what differentiates active from non-active post-MI patients.

Sense of coherence (SOC), a central construct of the salutogenic model of health, has been associated with health outcomes. This model was designed to explain improvement in one's location on the health-disease continuum [7], with SOC representing a person's confidence that they have the resources to cope with problems and challenges [8]. Previous research has reported a link between a strong SOC and reduced mortality from cancer and CVD [9,10], suggesting that SOC confers a form of resilience against disease. The explanatory pathway for this relationship remains unclear, although research has indicated that health behaviours may explain some of the association [10].

In a previous study, we found that post-MI patients with a stronger SOC were more likely to quit smoking than those with a weak SOC [11]. In the current study, we examined whether a similar relationship exists with post-MI physical activity. By elucidating which factors influence the adoption of important secondary prevention behaviours including physical activity, post-MI care can be improved. We therefore examined the predictive role of SOC in the long-term trajectory of LTPA in a community cohort of post-MI patients followed up for 13 years.

## Methods

### Study design and sample

Participants were drawn from the Israel Study of First Acute Myocardial Infarction, a multi-centre prospective cohort study of all patients ≤ 65 years admitted to hospital in central Israel with incident MI between February 1992 and February 1993.

From an initial sample of 1,521 patients enrolled in the parent study, representing 98% of all eligible MI survivors in the study period, 643 patients completed a questionnaire assessing SOC and were included in the current study. The remainder of the sample either declined to complete psychosocial questionnaires, following numerous medical examinations, or were excluded due to language barrier (the cohort included a high percentage of patients whose first language was not Hebrew)[12]. On average, non-respondents were slightly older (54.8 vs. 52.4 years), less educated (10 vs. 12 years of schooling), more likely to be female (22% vs. 15%) and unemployed pre-MI (34% vs. 12%), and had a higher prevalence of comorbid conditions (42% vs. 35%) compared with those providing psychosocial data. All participants provided written informed consent. The parent study was approved by the Ethics Committees of all medical centres involved (Wolfson, Holon; Sheba, Tel Hashomer; Tel Aviv Sourasky, Tel Aviv; Meir, Kfar Sava; Assaf Harofeh, Zerifin; Beilinson, Petach Tikvah; Hasharon, Petach Tikvah; and Laniado, Netanya) and ratified by the Institutional Ethics Committee of Tel Aviv University. The current study, which does not involve any additional patient contact, has been approved by the Institutional Ethics Committee of Tel Aviv University.

### Data collection

Inpatient and outpatient medical records, and data obtained through structured interviews, were used to ascertain demographics, socioeconomic status (SES) measures, cardiovascular disease (CVD) risk factors, MI characteristics and psychosocial factors. Interviews were conducted during initial hospitalization (T1), and subsequently 3 to 6 months (T2), 1 to 2 years (T3), 5 years (T4) and 10 to 13 years (T5) after MI.

#### Sociodemographic variables

SES data were self-reported at study entry and included the following measures: family income relative to the national average, education (years of schooling), ethnic origin (Israeli-born Jews, Mid-Eastern/North African, European/North American, others) and living with a steady partner.

#### Clinical variables

Cardiovascular risk factors, MI characteristics and disease severity indices were recorded at the index hospitalization. Obesity was defined as body mass index ≥ 30 kg/m^2^. Cigarette use was classified into current smoking vs. never or past smoking. Diabetes mellitus was defined as (a) fasting blood glucose ≥ 126 mg/dl on repeated measurements; (b) 2-hour blood glucose ≥ 200 mg/dl following glucose loading; or (c) use of insulin or oral hypoglycemic medication and a history consistent with diabetes. Hypertension was defined as systolic blood pressure ≥ 140 mm Hg, diastolic blood pressure ≥ 90 mm Hg, or use of antihypertensive medication. Dyslipidemia was defined based on the following criteria: (a) elevated total cholesterol (> 200 mg/dl); (b) elevated LDL cholesterol (> 100 mg/dl); (c) low HDL cholesterol (< 40 mg/dl for men, < 50 mg/dl for women); or (d) elevated triglycerides (> 150 mg/dl). Leisure time physical activity (LTPA) (regular, irregular or none) in the year preceding the index MI was self-reported.

Comorbidity was assessed by the Charlson index [13] and analyzed categorically (no comorbidity for 0 points, moderate comorbidity for 1 to 2 points, and severe comorbidity for 3 points or more). MI characteristics and severity indicators included infarct type and location and Killip class (categorised as ≥ 2 vs. 1). Self-rated health, a single item measure (5 = excellent health), was used to assess general health in the year preceding the MI [12].

#### Psychosocial factors

SOC and depression were assessed during index hospitalization. The Sense of Coherence scale [14], a 29-item questionnaire, measures personality resources for coping with stress and comprises the three constructs of comprehensibility, manageability and meaningfulness. Scores range from 29-203, with a higher score representing a stronger sense of coherence. This scale has been demonstrated as reliable and valid. A systematic review of 124 studies reported Cronbach's alpha ranges from 0.70 to 0.95 [15]. Depressive symptoms were measured using the Beck Depression Inventory (BDI), a 21-item questionnaire widely used to assess subclinical and clinical depression [14,16]. The Hebrew versions of these questionnaires were previously demonstrated as having high reliability in this cohort (all Cronbach's α ≥ 0.85) [17].

#### Outcome variable

Follow-up was initiated at the date of index MI and continued until 31 December, 2005 (median [IQR], 13.2 [12.0-13.5] years; loss to follow-up < 2%). LTPA during follow-up was assessed by self-report questionnaire administered on four occasions (T2-T5). At each interview, patients were asked to report their current participation in LTPA activities, including walking, cycling, swimming, gardening, going to the gym and team sports and to report the average frequency (times per week) and duration of each session. These responses were summarised by a senior cardiologist (Y.D.) into 3 groups: inactive, irregularly active and regularly active patients. Regular physical activity was defined as at least three 30 minute sessions per week, according to published guidelines [18]. Physical activity reported at a lower frequency was defined as irregular, and individuals who reported no activity were defined as inactive. While this scale, developed for the study, has not been validated or tested for reliability over repeated administrations, it was shown to predict post-MI mortality in a previous study [5].

### Statistical analysis

Analyses were performed using SPSS version 19.0 (SPSS Inc., Chicago, IL). Baseline characteristics across SOC tertiles were analyzed using ANOVA for continuous variables and chi-square test for categorical variables.

The predictive role of SOC in long-term trajectory of LTPA after MI was assessed with generalized estimating equations (GEE) [19], utilizing a cumulative logit link function for multinomial distribution and an exchangeable correlation structure. LTPA (categorized as regular, irregular, or none), reported 4 times during follow-up (T2-T5), was treated as an ordinal outcome. GEE was selected for analysis because of the longitudinal nature of the dataset. Longitudinal data are characterized by the fact that repeated measures for a subject tend to be correlated. The GEE method takes into account this within-subject correlation, assuming that the outcomes from different individuals are independent, whereas outcomes coming from the same individual are correlated. To facilitate the interpretation of the results, all GEE parameter estimates were exponentiated and presented as odds ratios (OR) and 95% confidence intervals (CI). Age- and sex-adjusted models were initially examined, followed by hierarchical models incorporating multilevel SES measures, CVD risk factors (including LTPA before MI), MI characteristics and disease severity indices, and depression measured at study entry. Missing values in any of the variables evaluated did not exceed 2%. Reports of LTPA during follow-up were missing in 10% at T2, 15% at T3, and < 1% at T4 and T5, among survivors.

## Results

Baseline patient characteristics across SOC tertiles are shown in Table 1. Mean SOC score was 145.7 (SD 25). Patients with a strong SOC were more likely to be employed pre-MI, more highly educated, and of European or North American extraction and were less likely to be current smokers than those with a weak SOC. Additionally, high SOC scorers had significantly fewer depressive symptoms and were more likely to be regularly active pre-MI. There was no difference in the presence of cardiac risk factors other than smoking or in disease severity markers according to SOC, although high scorers rated their health as better than low scorers.

**Table 1:** Baseline characteristics by sense of coherence categories

Characteristic	SOC Tertiles			*P*
	
	Lower	Middle	Upper	
	≤ 138	139-158	> 158	
	(n = 219)	(n = 216)	(n = 208)	
**Sociodemographics**				

Age (years); mean (SD)	52.1 (8.8)	52.7 (8.1)	52.3 (8.9)	0.76

Male	82%	88%	86%	0.17

Ethnic origin				0.008
Israeli Jews	34%	33%	42%	
Mid-Eastern/North African	33%	31%	17%	
European/North American	31%	33%	39%	
Others	2%	3%	2%	

Education (years); mean (SD)	11.0 (3.6)	12.6 (3.8)	12.5 (3.5)	< 0.001

Relative income				< 0.001
Above-average	26%	39%	44%	
Average	31%	32%	32%	
Below-average	43%	29%	24%	

Pre-MI employment	81%	89%	91%	0.004

Living with a steady partner	87%	91%	91%	0.24

**Cardiovascular Risk Factors**				

Hypertension	36%	37%	36%	0.99

Diabetes	21%	19%	17%	0.55

Dyslipidemia	37%	38%	40%	0.74

Smoking				< 0.001
Never	27%	19%	32%	
Past	17%	22%	31%	
Present	55%	58%	38%	

Obesity	18%	14%	17%	0.49

Pre-MI LTPA				0.01
Regularly active	30%	32%	40%	
Irregularly active	13%	23%	17%	
Inactive	57%	45%	43%	

Depression score; mean (SD)	10.9 (8.1)	6.4 (5.4)	3.9 (4.6)	< 0.001

**MI Characteristics & Disease Severity Indices**				

Killip class > 1	18%	22%	16%	0.24

Q-wave MI	78%	83%	79%	0.34

Anterior MI	43%	49%	37%	0.04

Charlson comorbidity index				0.18
0 points	66%	59%	69%	
1 to 2 points	31%	38%	27%	
≥ 3 points	3%	2%	3%	

Self-rated health; mean (SD)	3.5 (1.2)	3.7 (1.0)	3.9 (1.0)	0.001

SOC, sense of coherence; SD, standard deviation; MI, myocardial infarction; SES, socioeconomic status; LTPA, leisure time physical activity.

**Figure 1:**
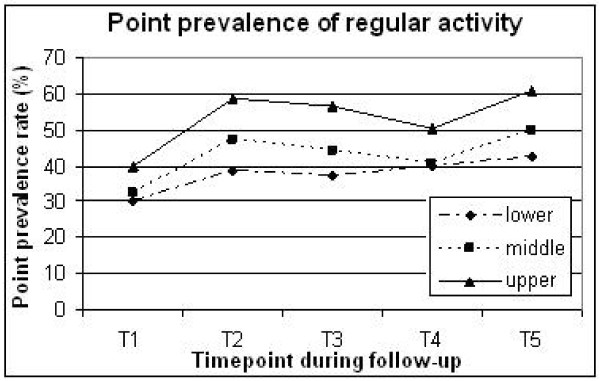
**Percentage of patients regularly engaged in leisure time physical activity at different time points throughout the study across sense of coherence tertiles (upper, middle and lower)**. T1, baseline (pre-MI); T2, 3-6 months; T3, 1-2 years; T4, 5 years; T5, 10-13 years post-MI.

**Figure 2:**
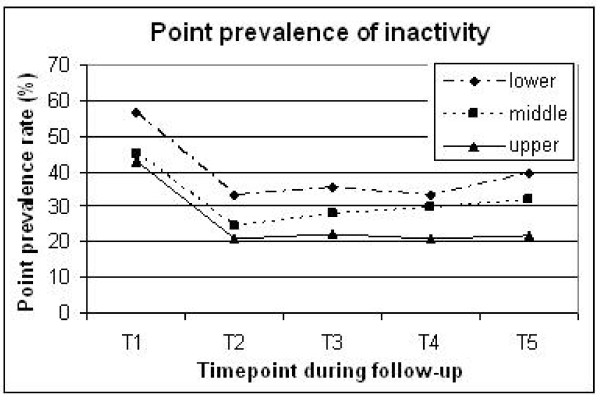
**Percentage of inactive patients at different time points throughout the study across sense of coherence tertiles (upper, middle and lower)**. T1, baseline (pre-MI); T2, 3-6 months; T3, 1-2 years; T4, 5 years; T5, 10-13 years post-MI.

SOC clearly predicted LTPA throughout follow-up. Point prevalence of regular activity in the lower versus upper tertiles was 37-42% versus 50-60% during T2-T5 (Figure 1). Inactivity was reported in 33-39% of the lower SOC tertile, versus 20-22% of the upper tertile (Figure 2).

The GEE-derived OR (95% CI) for decreasing LTPA trajectory are presented in table 2. In an age- and sex-adjusted model, patients in the lowest SOC tertile had almost twice the odds (OR,1.99; 95% CI,1.52-2.60) of decreasing their engagement in LTPA during follow-up as those in the highest tertile. While this association was somewhat reduced on adjustment for SES, ethnicity, cardiac risk factors, disease severity measures and depression, it remained significant, with a 38% increased odds of decreasing LTPA in the lower versus upper tertiles (OR,1.38; 95% CI 1.02-1.85). The association between SOC and LTPA was further examined by timepoint (Table 3); after multivariable adjustment, the strongest association was seen after 10-13 years.

**Table 2:** Odds ratios (95% confidence intervals) for decreasing level of engagement in leisure time physical activity after myocardial infarction associated with sense of coherence categories^a^

	SOC Tertiles			
**Adjustment**	**Lower**	**Middle**	**Upper**	***P *for trend**

Age and sex	1.99 (1.52-2.60)	1.54 (1.18-2.02)	1	< 0.001

Model 1	1.69 (1.28-2.24)	1.52 (1.15-2.00)	1	< 0.001

Model 2	1.53 (1.16-2.02)	1.43 (1.09-1.88)	1	0.003

Model 3	1.38 (1.02-1.85)	1.35 (1.02-1.79)	1	0.03

MI, myocardial infarction; LTPA, leisure time physical activity; SES, socioeconomic status; SOC, sense of coherence

^a^Estimates are derived from GEE analyses with LTPA as an ordinal outcome (classified as regular, irregular, or none).

Model 1: Adjusted for age, sex, ethnicity, education, relative income, pre-MI employment, and living with a steady partner.

Model 2: Model 1 plus hypertension, diabetes, dyslipidemia, smoking, obesity, pre-MI leisure time physical activity, Killip class, Q-wave MI, anterior MI, Charlson comorbidity index, and self-rated health.

Model 3: Model 2 plus depression.

**Table 3:** Odds ratios (95% confidence intervals) for decreasing level of engagement in leisure time physical activity after myocardial infarction associated with sense of coherence categories, by timepoint during follow-up^a^

LTPA At Different Time Periods During Follow Up				
**Adjustment**	**SOC Tertiles**			

3-6 months (n = 579)	**Lower**	**Middle**	**Upper**	***P *for trend**

Age and sex	2.13 (1.45-3.12)	1.48 (1.00-2.17)	1	< 0.001

Model 1	1.84 (1.23-2.75)	1.45 (0.97-2.15)	1	0.003

Model 2	1.62 (1.06-2.47)	1.30 (0.87-1.97)	1	0.02

Model 3	1.45 (0.93-2.27)	1.27 (0.83-1.93)	1	0.10

1-2 years (n = 537)				

Age and sex	2.09 (1.41-3.11)	1.60 (1.07-2.39)	1	< 0.001

Model 1	1.90 (1.26-2.86)	1.62 (1.07-2.44)	1	0.002

Model 2	1.65 (1.07-2.55)	1.47 (0.95-2.28)	1	0.02

Model 3	1.42 (0.89-2.26)	1.42 (0.91-2.21)	1	0.14

5 years (n = 612)				

Age and sex	1.66 (1.15-2.39)	1.54 (1.07-2.21)	1	0.007

Model 1	1.46 (1.00-2.13)	1.52 (1.05-2.20)	1	0.05

Model 2	1.34 (0.90-1.99)	1.51 (1.02-2.24)	1	0.15

Model 3	1.21 (0.79-1.86)	1.39 (0.93-2.07)	1	0.37

10-13 years (n = 553)				

Age and sex	2.16 (1.45-3.22)	1.64 (1.10-2.44)	1	< 0.001

Model 1	1.89 (1.25-2.87)	1.67 (1.11-2.52)	1	0.001

Model 2	1.89 (1.22-2.91)	1.55 (1.01-2.38)	1	0.004

Model 3	1.87 (1.17-3.00)	1.46 (0.94-2.27)	1	0.009

MI, myocardial infarction; LTPA, leisure time physical activity; SES, socioeconomic status; SOC, sense of coherence

^a^Estimates are derived from GEE analyses with LTPA as an ordinal outcome (classified as regular, irregular, or none).

Model 1: Adjusted for age, sex, ethnicity, education, relative income, pre-MI employment, and living with a steady partner.

Model 2: Model 1 plus hypertension, diabetes, dyslipidemia, smoking, obesity, pre-MI leisure time physical activity, Killip class, Q-wave MI, anterior MI, Charlson comorbidity index, and self-rated health.

Model 3: Model 2 plus depression

Patients with a weaker SOC exhibited less dramatic changes in LTPA levels after MI compared with those with a stronger SOC. While among strong SOC patients point prevalence of regular activity improved by approximately 20% during follow up, it improved by around 10% in the lower SOC patients (Figure 1).

## Discussion

In this cohort of post-MI patients, the predictive role of SOC in long-term trajectory of LTPA was evident, even after controlling for sociodemographic and psychological factors and disease severity. Weak SOC was consistently associated with lower engagement in LTPA throughout follow-up. SOC was associated with income, educational level, employment status, self-rated health and depressive symptoms, matching the findings from a healthy population survey [20], and indicating that SOC reflects personal, social and economic resources.

Previous research has investigated the role of SOC in health outcomes and behaviours. The European Prospective Investigation into Cancer (EPIC) study reported a 30% reduction in both all-cause and cardiovascular mortality associated with a strong SOC, independent of age, sex, and prevalent chronic disease [9]. This link suggests that SOC confers a form of resilience against disease, however the explanatory pathway for the SOC-mortality relationship remains unclear. In a follow-up of the EPIC, lifestyle choices including diet, smoking and physical activity, and SES explained 23% of the association between SOC and mortality [10]. Since lifestyle behaviours were measured at a single time point, their true contribution may be even greater. The current study measured physical activity on 5 separate occasions over a 13 year period and found a significant association between SOC at baseline and LTPA trajectory.

Evidence for the link between SOC and health behaviours has been reported in several studies, including in healthy college students [21] and hypertensive patients [22]; SOC has previously been associated with smoking [22,23] and oral health behaviours [24-26], as well as physical activity [10,23,24,27]. In a British survey of 18,000 adults, participants with the strongest SOC were 28% less likely to be current smokers and 36% less likely to be physically inactive than those with the weakest, independent of sociodemographics [23], although this study was based on a 3-item assessment of SOC rather than the full 29-item questionnaire used in the present study. In a student cohort followed up for 3 years, frequency of physical activity was related to strength of SOC [27]. A prospective study of middle-aged Finnish men found that differences in LTPA depended on SOC [28]. However, these studies were cross-sectional [23,24], involved small samples [27], or assessed SOC several years after physical activity [28] therefore providing limited evidence of the predictive role of SOC in physical activity.

The relationship between SOC and health behaviours has rarely been examined in MI patients. A small-scale study tackled this issue, and found that post-MI patients who chose to participate in an exercise-based rehabilitation programme had significantly higher SOC scores at baseline than those who declined to take part [29]. Additionally, a discriminant analysis showed that SOC score successfully differentiated attendees from non-attendees. The current study examined the predictive role of SOC in engagement in physical activity in a much larger sample, controlled for a wide range of confounding variables, and had a follow-up of 13 years. We focused on leisure time physical activity since, although cardiac rehabilitation programmes are indispensable in post-MI recovery, long-term maintenance of physical activity is key to secondary prevention.

Our finding that baseline SOC score was significantly associated with LTPA after 10-13 years suggests that a strong SOC may be involved in the maintenance of health behaviours. Figure 1 illustrates that LTPA prevalence increased in all groups after MI, although the increase was steeper in strong SOC patients. The problem encountered, as with all health behaviours, is the gulf between adoption and maintenance, indeed Leung and colleagues found in a cohort of patients with coronary artery disease that while exercise behaviour increased after discharge from hospital, it had decreased after 18 months [30]. It is therefore interesting to note that while many of our post-MI cohort increased their activity level after MI, no doubt in many cases on medical advice, it was the individuals with a weak SOC who had much higher odds of decreasing levels of activity 10-13 years later. It should also be noted that at the last interview (T5), less than 1% of LTPA data were missing, therefore a more accurate picture of activity habits was available compared to earlier timepoints which had 10-15% missing data.

A strong SOC was additionally associated with pre-MI LTPA habits, perhaps indicating that these individuals led a healthier lifestyle to begin with. We may then have expected that high SOC patients would remain consistently ahead of their lower SOC counterparts in the activity stakes. The fact that LTPA not only remained more prevalent, but increased at a higher rate post-MI in the higher compared to lower SOC groups, suggests that SOC may be related both to health behaviours in general, and to secondary prevention behaviours as an adaptive coping mechanism after illness.

### Possible mechanisms

The salutogenic model was designed to explain improvement in one's location on the health-disease continuum [7]. Antonovsky hypothesized that individuals with a strong SOC would engage in adaptive health behaviours more often than those with a weak SOC [14]; however he also suggested that strength of SOC has direct physiological consequences affecting health, an early nod to psychoneuroimmunology [31].

A strong SOC has been associated with reduced mortality [9,10], suggesting that SOC confers a form of resilience against disease. Health behaviours, including physical activity, may be one of the ways in which SOC influences health. SOC level rests on the presence or absence of generalized resistance resources [8]-these may include material and financial resources (eg. money for gym membership or sports equipment), social support (a supportive partner or friend with whom to engage in exercise), flexibility (the ability to make changes to one's daily routine and adapt to change), knowledge and physical health status. Patients with a strong SOC in our cohort possessed many of these resources which may facilitate achievement of health goals. Furthermore, high SOC may improve one's ability to identify available resources [15], improving the chances of choosing an appropriate coping strategy from among these resources.

According to Antonovsky, SOC is as an adaptive dispositional orientation, with the three components of SOC determining how an individual adapts to a stressful situation [14]. Surtees and colleagues further reported that a weak SOC was associated with a slower adaptation to adverse events [32]. Adaptive capacity may then be the most important element of SOC in post-MI patients, since recovery and rehabilitation require lifestyle changes. Perception of the condition as manageable, comprehensible and meaningful allows the individual to perceive the situation as a challenge, and to accurately identify and make use of resources in his environment in order to adapt and overcome this challenge [14]. This may include making significant changes to daily life. On the contrary, perception of the situation as overwhelming, incoherent and exceeding one's resources may reduce adaptive capacity, making lifestyle changes difficult. In the same way that lower SOC individuals were less likely to quit smoking post-MI [11], so they may also find it harder to adopt and maintain physical activity habits, due to reduced resources and lower adaptive capacity.

Finally, it has been suggested that individuals with a strong SOC may more accurately identify the nature of a situation [33]. In the context of illness this may translate to a better comprehension of the influence of behaviour on health outcomes, which could motivate behaviour change.

### Methodological considerations

Several limitations of our research should be considered when interpreting the results of this study. Participants were relatively young survivors of MI, therefore results may not be generalizable to older patients. LTPA was self-reported, which may introduce misclassification bias. SOC was measured just once, during initial hospitalization, which may cause a substantial underestimation of the true association between SOC and LTPA after MI. Antonovsky described SOC as a stable trait, "a global orientation that is pervasive and enduring" [8], formed by childhood experiences and reinforced by life experiences. Indeed test-retest correlations show considerable stability, e.g. 0.54 over a 2-year period among retirees [7]. While traumatic events including illness can temporarily modify the SOC, it is expected to return to normal or 'bounce back' [14]; in fact SOC scores in our post-MI cohort were previously reported as being similar to population scores, implying that even the serious event of MI may not significantly alter SOC [34]. However, some research indicates that SOC may vary with time, for example in a cohort of accident victims, SOC decreased following trauma and remained lowered after 12 months [35] and in CABG patients, 41% showed changes in their SOC during a 1 year follow-up [36].

The study benefitted from repeated assessment of LTPA over 13 years and parallel assessment of multiple confounding factors. There are some indications that depression may be associated with post-MI LTPA [37] therefore we took this variable into consideration as a confounding factor.

## Conclusion

SOC was found to predict LTPA trajectory in a cohort of MI survivors. The primary use of this finding is in identifying high-risk MI survivors, with SOC representing a summary measure of personal, social, psychological and economic resources. SOC is easy to ascertain during hospitalization and can help to identify patients who are less likely to incorporate physical activity in their daily life, who may require additional help in adopting preventive health behaviours, which can dramatically improve post-MI prognosis. These findings have implications not only for secondary prevention of cardiovascular disease but for other illnesses and may be applicable to health promotion in general.

## Declaration of Competing interests

The authors declare that they have no Competing interests.

## Authors' contributions

YG conceived of the study, participated in the study design, performed statistical analyses and reviewed the manuscript. YD participated in the study conception, acquired data and reviewed the manuscript. VM participated in the study conception and design, interpreted the data and drafted the manuscript. All authors read and approved the final manuscript.

## Funding

This work was supported in part by the Israel National Institute for Health Policy and Health Services Research (grant number r/89/2008 to Drs. Drory and Gerber), and by the Environment and Health Fund, Jerusalem, Israel (grant award number RGA0904 to Dr. Gerber), which had no role in study design, data acquisition, writing of the manuscript or decision to submit for publication.

## Pre-publication history

The pre-publication history for this paper can be accessed here:

http://www.biomedcentral.com/1471-2458/11/708/prepub
